# Pembrolizumab-induced myocarditis with complete atrioventricular block and concomitant myositis in a metastatic bladder cancer patient: a case report and review of the literature

**DOI:** 10.1186/s13256-024-04397-3

**Published:** 2024-02-22

**Authors:** R. Saad, A. Ghaddar, R. M. Zeenny

**Affiliations:** 1 Department of Pharmacy, American University of Beirut Medical Center, Riad El-Solh, P.O. Box 11-0236, Beirut, 1107 2020 Lebanon; 2 INSPECT-LB (Institut National de Santé Publique, d’Épidémiologie Clinique et de Toxicologie-Liban), Beirut, Lebanon

**Keywords:** Immune checkpoint inhibitor, Immune-related adverse event, Immune-checkpoint inhibitor-associated myocarditis, Cardiotoxicity, Myositis, Pembrolizumab, PD-1, Cardio-oncology

## Abstract

**Background:**

The cardiovascular system is among the least systems affected by immune-related adverse events. We report a rare life-threatening case of pembrolizumab-induced myocarditis with complete atrioventricular block and concomitant myositis in a metastatic bladder cancer patient.

**Case presentation:**

An 82-year-old Caucasian female with invasive urothelial carcinoma, started on first-line pembrolizumab, was admitted four days after receiving her second dose for severe asthenia, diffuse muscle aches, neck pain, and lethargy. In the emergency department, she had several episodes of bradycardia reaching 40 beats per minute associated with general discomfort and fatigue. Electrocardiography showed a third-degree atrioventricular heart block, while the patient remained normotensive. Cardiac damage parameters were altered with elevated levels of creatine phosphokinase of 8930 U/L, suggestive of immune checkpoint inhibitor-induced myositis, and troponin T of 1.060 ng/mL. Transthoracic echocardiography showed a preserved ejection fraction. Pembrolizumab-induced myocarditis was suspected. Therefore, treatment was initiated with high-dose glucocorticoids for 5 days, followed by a long oral steroid taper. A pacemaker was also implanted. Treatment resulted in the resolution of heart block and a decrease in creatine phosphokinase to the normal range.

**Conclusion:**

Life-threatening cardiac adverse events in the form of myocarditis may occur with pembrolizumab use, warranting vigilant cardiac monitoring. Troponin monitoring in high-risk patients, along with baseline echocardiography may help identify this complication promptly to prevent life-threatening consequences.

## Background

In recent years, immune checkpoint inhibitors (ICIs) have transformed the landscape of cancer treatment [1]. Malignant cells evade the recognition and destruction by the immune system by exploiting immune checkpoint receptors, such as the cytotoxic T lymphocyte-associated antigen 4 (CTLA-4), programmed cell death protein 1 (PD-1), and programmed cell death ligand 1 (PD-L1). Immune checkpoint inhibitors are drugs designed to disrupt these interactions, reactivating the immune system, and generating potent and long-lasting antitumor responses [2, 3]. Up to now, the US Food and Drug Administration (FDA) has approved several ICIs for cancer treatment, including one CTLA-4 inhibitor (ipilimumab), three PD-1 inhibitors (nivolumab, pembrolizumab, cemiplimab), and three PD-L1 inhibitors (atezolizumab, avelumab, durvalumab) [4]. Although ICIs are overall well tolerated, several immune-related adverse events (irAEs) have been reported, which are due to the suppression of immune regulation and inflammatory reactions against the normal tissues [5]. These include hepatitis (< 10%), colitis (1–2%), endocrine disorders (1–6%), and skin lesions (30–40%) [5]. The cardiovascular system is among the least systems affected by irAEs with pericarditis, myocardial fibrosis, pericardial effusion, and myocarditis being some of the possible side effects [6]. Although very rare, myocarditis is being increasingly reported with the use of ICIs and has been estimated to occur in 0.06–1% of clinical trial patients receiving immunotherapy [6]. Although data on the presentation, diagnosis, and outcomes are limited, myocarditis often appears as a fulminant and severe side effect of ICIs [7]. Hence, a high degree of awareness is essential to detect this rare complication in patients on ICI therapy to initiate appropriate treatment promptly.

Pembrolizumab is uncommonly associated in the literature with immune checkpoint inhibitor-associated myocarditis (ICIM) when compared to other ICIs [8–10], with a low incidence of the life-threatening nature of this side effect [7, 9]. Moreover, ICIM is more described in melanoma and non-small cell lung cancer patients, while urothelial carcinoma is rarely mentioned [8, 9]. Only three cases of pembrolizumab-induced myocarditis with complete AV block in bladder cancer patients were identified [10–12]. We report a rare life-threatening case of pembrolizumab-induced myocarditis with a complete heart block and concomitant myositis in an 82-year-old bladder cancer patient.

## Case presentation

An 82-year-old Caucasian female with a history of hypertension on candesartan, diabetes mellitus type 2 poorly controlled on glimepiride, repaglinide, and vildagliptin, dyslipidemia on fenofibrate, and no history of auto-immune disease, was treated with her second dose of the first-line pembrolizumab for invasive poorly differentiated urothelial carcinoma.

The patient was diagnosed with papillary transitional cell carcinoma of the bladder in 2009 for which she underwent a transurethral resection (TURBT). This was followed by another resection in 2013, followed by gemcitabine chemotherapy, then several resections in 2015, followed by chemoradiation with cisplatin and gemcitabine. She underwent resections in 2016 and 2018, followed by intravesical instillation of mitomycin C. She presented in July 2019 for an increased size caruncle which was resected. The tissue was positive for invasive poorly-differentiated urothelial carcinoma, expressing PD-L1 in 50% of the tumor cells. A CT of the abdomen and pelvis showed enlarged bilateral inguinal lymph nodes suggestive of metastatic disease. Subsequently, a lymph node biopsy confirmed the diagnosis of metastatic poorly differentiated urothelial carcinoma stage IV A (AJCC 8th Edition) [13] for which she was started on first-line immunotherapy with pembrolizumab 200 mg intravenous drip every 3 weeks. She received her first cycle in September 2019 without complications, presented for her second cycle 21 days later with mild fatigue, and received her second dose. Four days later she contacted her oncologist complaining of severe neck pain and was advised to present to the emergency department.

**Fig. 1:**
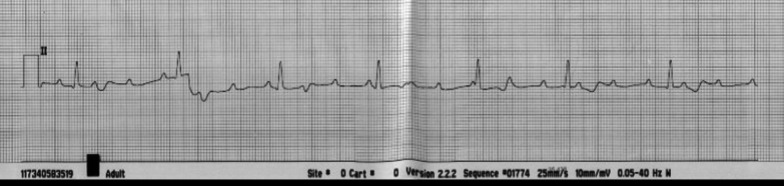
Electrocardiogram (ECG) on the day of admission to the emergency department: ECG showing a complete atrioventricular block with a ventricular escape rhythm

The patient was admitted to the hospital 25 days after the initial dose of pembrolizumab for severe asthenia, diffuse muscle aches, neck pain, and lethargy. She denied chest pain, orthopnea, or paroxysmal nocturnal dyspnea. In the ED, she had several episodes of bradycardia reaching 40–46 beats per minute associated with general discomfort and fatigue. The initial workup revealed a third-degree atrioventricular (AV) heart block in the electrocardiogram (ECG; Fig. 1) while the patient was normotensive. Moreover, cardiac damage parameters were altered with elevated levels of creatine phosphokinase kinase (CPK) of 8930 U/L [normal range (NR) 20–165 U/L], suggestive of severe rhabdomyolysis, and troponin T (TnT) of 1.060 ng/mL (NR ≤ 0.030 ng/mL) (Table 1). Additionally, her serum potassium was elevated (6.5 mmol/L, [NR 3.5–5.1 mmol/L]). Due to these alterations, a transthoracic echocardiogram (TTE) was performed urgently, showing a preserved left ventricular ejection fraction (LVEF). Urine chemistry showed positive myoglobin. Serum creatinine, as well as liver function tests, were within normal limits. The chest radiograph showed a clear lung field. Acetylcholine receptor binding antibodies’ (AChR-Ab) level was positive borderline at 0.31 nmol/L (NR < 0.25 nmol/L; Borderline 0.25–0.4 nmol/L) and antibodies to muscle-specific tyrosine kinase (MuSK-Ab) were negative.

**Table 1:** Summary of relevant clinical parameters upon admission to the emergency department

	D0 (ED)	D1	D2	D3	D4							
CPK (IU/L)	8930	6220	2630	1929	1547	1200	640	536	260	240	107	53
TnT (ng/mL)	1.06										0.339	
ECG	Complete AV block											
TTE	LVEF > 70%									LVEF > 70%		
BP (mmHg)	165/41	155/40	169/61	162/68	150/86	142/76	130/54	154/79	164/69	153/67		
HR (beats/min)	40	41	74	96	88	95	68	79	63	83		
K (mmol/L)	6.5	5.2	4.5	3.8	3.9	4.2	3.8	3.9	4.4	4.3	4.6	

*ED* Emergency Department, *CPK* creatine phosphokinase, *TnT* troponin T, *ECG* electrocardiogram, *TTE* transthoracic echocardiogram, *LVEF* left ventricular ejection fraction, *SBP* blood pressure, *HR* heart rate, *K* serum potassium (mmol/L), *IU/L* international unit/liter

Given the elevation of markers of cardiac injury and ECG changes with the absence of other cardiac syndromes or infectious signs, alongside the history of immunotherapy and concomitant myositis, pembrolizumab-induced myocarditis was suspected. An endomyocardial biopsy could not be performed due to the invasive nature of the procedure and the risk of potential acute and chronic complications. Other etiologies of myocarditis (that is, viral myocarditis, giant cell myocarditis, eosinophile myocarditis, endomyocardial fibrosis, sarcoidosis) were excluded. The elevated CPK was linked to pembrolizumab-induced myositis rather than fibrate-induced rhabdomyolysis, knowing the patient was maintained on fenofibrate for several years. It was however judged preferable to discontinue the medication. The borderline positive AChR-Ab was suggestive of possible *myasthenia gravis*. The patient was started on a dopamine drip and treatment was initiated with high-dose glucocorticoids (1 mg/kg/day of intravenous methylprednisolone) for 5 days. The patient was then transitioned to oral prednisone followed by a long taper over five weeks. A pacemaker was implanted 3 days later. A follow-up ECG showed complete recovery of sinus rhythm and heart rate.

## Discussion

Pembrolizumab is a PD-1 immune checkpoint inhibitor that has significantly increased overall survival in a broad array of cancer types, including melanoma, non-small cell lung cancer, renal cell carcinoma, and microsatellite instability-high or mismatch repair-deficient cancer [14]. Although recognized as an uncommon adverse reaction, ICIM may result in poor outcomes [15]. We report in this article a case of life-threatening myocarditis with a complete AV block occurring in an 82-year-old female four days after receiving her second dose of pembrolizumab for invasive urothelial carcinoma. We reviewed the pertinent literature for pembrolizumab and other ICIs-induced myocarditis.

### Incidence of myocarditis and fatality outcomes

Myocarditis associated with ICIs is considered a relatively rare adverse event, with a reported incidence of 0.04–1.14% [16, 17] increasing up to 2.4% with combination therapy [7]. However, it is associated with poorer outcomes when compared to other immune-related adverse events, with higher mortality rates ranging between 25 and 50% [7, 8, 15, 18, 19].

Pembrolizumab is associated in the literature with a low incidence of ICIM when compared to other immune-checkpoint inhibitors [8–10], as well as a low incidence of the life-threatening nature of this side effect [7, 9]. In our patient, on Naranjo's causality assessment scale, the adverse event was 6 indicating a “probable” reaction to pembrolizumab (Table 2) [20]. Among the 315 patients with ICIM identified in a post-marketing surveillance study by Fan *et al.*, nivolumab monotherapy had the highest number of case reports with 125 cases (39.6%), followed by the combination of ipilimumab plus nivolumab with 73 cases (23.1%). As for pembrolizumab monotherapy, there were 69 cases reported (29.90%) [9]. In a retrospective study of data from eight clinical centers by Mahmood *et al.*, of 35 patients who had myocarditis, 12 were receiving combinations of anti-CTLA-4 and anti-PD1/PD-L1, 11 were on pembrolizumab monotherapy and 7 on nivolumab monotherapy [7]. On the other hand, the analysis of the incidence of major cardiac events (MACE) showed that 44% of the cases were related to nivolumab, versus 13% with pembrolizumab. In the pharmacovigilance study conducted by Fan *et al.* using the Food and Drug Administration’s Adverse Event Reporting System (FAERS), the combination of ipilimumab plus nivolumab was significantly associated with myocarditis fatality (65.75%), while nivolumab was the monotherapy mostly correlated with a risk of myocarditis death (50.4%) [9].

**Table 2:** Naranjo algorithm—adverse drug reaction probability scale

Question	Yes		Do not know	Score
1. Are there previous conclusive reports on this reaction?	+ 1	0	0	+ 1
2. Did the adverse event appear after the suspected drug was administered?	+ 2	− 1	0	+ 2
3. Did the adverse event improve when the drug was discontinued or a specific antagonist was administered?	+ 1	0	0	+ 1
4. Did the adverse event reappear when the drug was readministered?	+ 2	− 1	0	0
5. Are there alternative causes that could on their own have caused the reaction?	− 1	+ 2	0	+ 2
6. Did the reaction reappear when a placebo was given?	− 1	+ 1	0	0
7. Was the drug detected in blood or other fluids in concentrations known to be toxic?	+ 1	0	0	0
8. Was the reaction more severe when the dose was increased or less severe when the dose was decreased?	+ 1	0	0	0
9. Did the patient have a similar reaction to the same or similar drugs in any previous exposure?	+ 1	0	0	0
10. Was the adverse event confirmed by any objective evidence?	+ 1	0	0	0
	Total score: 6			

Our patient was receiving pembrolizumab as a first-line treatment for invasive urothelial carcinoma, which is not commonly associated with ICIM in the literature where melanoma and non-small cell lung cancer appears to be more common [8, 9]. Only three cases of pembrolizumab-induced myocarditis with complete AV block in bladder cancer patients were identified [10–12]. The cases describe a pembrolizumab-induced *myasthenia gravis* [10] and myositis [11] followed a few days later by a complete AV block that resulted in death despite aggressive treatment. Similar to our case, the immune-related adverse event occurred in two elderly patients, early in the treatment course. Cardiac biomarkers were elevated, however, ECG was initially normal in the case described by Takai *et al.* [10], while a wide QRS complex was identified in the case described by Matsui *et al.* [11] Hellman *et al.* [12] reported a case of myocarditis with a second-degree AV block along with myositis in a 42-year old bladder cancer patient treated with the combination of pembrolizumab and epacadostat.

### Patients’ characteristics

Myocarditis seems to predominantly occur in elderly patients which aligns with our case, with males however being affected more than female patients [7, 9, 17]. Among the 315 patients with ICIM described by Fan *et al.* [9] 51.11% were above 65 years of age and 58.41% were men. Advancing age is an important risk factor for cancer [21]. It is to note that safety data on the use of ICIs in elderly patients is still limited, due to insufficient enrollment in clinical trials [22]. Underlying auto-immune disease, pre-existing cardiovascular disease, and diabetes mellitus might be risk factors for ICIM [6]. According to Mahmood *et al.*, myocarditis cases had a higher prevalence of diabetes mellitus, sleep apnea, and a higher body mass index [7]. Our patient had uncontrolled type 2 diabetes.

### Clinical presentation

The clinical presentation of ICIM can vary from asymptomatic raises in cardiac biomarkers to life-threatening fulminant decompensation, which is the most commonly reported in the literature [6]. This report describes an uncommon case of pembrolizumab-induced myocarditis with a complete atrioventricular block with the preservation of LVEF. In comparison to the 8 cases of pembrolizumab-inducted myocarditis presented in the systematic review by Atallah-Yunes *et al.*, four cases had a complete AV block, one of them only with a preserved LVEF. None of the cases occurred in a bladder cancer patient [28]. Out of the 35 cases of ICIM presented by Mahmood *et al.*, only 3 experienced a complete heart block with no specification of the implicated agent. 38% of those who developed MACEs had normal LVEF [7].

In this case report, myocarditis was also associated with myositis and borderline positive AChR-Abs. According to Palaskas *et al.* in a review article on ICIM, the presence of other immune-related adverse events increases the possibility of ICIM in patients presenting with cardiac symptoms [23]. Myositis and *myasthenia gravis* are commonly associated with ICIM [24]. The suggested explanation is shared antigens between cardiac muscle and skeletal muscles compared with other tissues [23]. Among the systematic review of the 43 published cases of ICIM by Atallah-Yunes *et al.*, 29% of the patients had concomitant myositis [28].

Another interesting finding is that myocarditis with ICI occurs early in the treatment course with ICIs, which aligns with our case where it developed 25 days after treatment initiation, following the second dose of pembrolizumab. This is consistent with the finding of the FAERS database analysis which describes a median time to onset of myocarditis of 23 days [interquartile range (IQR) 14–55 days] [9]. In the VigiBase study, 64% of the patients who had information available developed ICIM after the first or second dose of ICI. However, late presentations have also been reported in the literature [17]. Clinicians should maintain a high level of clinical suspicion of this serious adverse event, notably in elderly patients after the first doses of immunotherapy, although diagnosis should also be considered in patients with a long history of treatment with ICIs.

### Diagnostic tests

The two most common laboratory tests that may initially be suggestive of myocarditis are elevated serum troponin and natriuretic peptide levels [25]. In the cohort study of Mahmood *et al.*, almost all myocarditis cases had a troponin elevation (94%), the degree of troponin elevation (initial level, peak, and discharge level) being a predictor of adverse events, alongside an abnormal ECG (89%) [7]. A level of Troponin T ≥ 1.5 ng/mL upon discharge was a poorer prognosis, with a fourfold increased risk of MACE. However, a depressed LVEF was not a precondition for serious adverse cardiovascular events, in comparison to non-immune therapy-related myocarditis [7]. Our patient had a mildly elevated troponin T and an abnormal ECG, with a preserved LVEF. Serum troponin is an inexpensive test that is commonly available, a rise generally suggesting myocyte death [26]. Consequently, because the onset of myocarditis often occurs around the first or second dose of ICIs, checking troponin levels at baseline and each cycle may be of value, especially in high-risk patients, notably the elderly. An elevated value would warrant an urgent referral to cardiology for further evaluation, in the light of suspected ICI-induced myocarditis, potentially preventing a fatal outcome.

### Treatment

To date, discontinuation of ICIs and immunosuppression with glucocorticoids represent the cornerstone of the management of ICIM. The ASCO clinical practice guidelines for the management of irAE suggest the initiation of 1 mg/kg daily of either intravenous or oral prednisone or equivalent followed by a taper over 4–6 weeks [27]. Our patient was treated with 1 mg/kg per day of methylprednisolone followed by oral prednisone tapered over 6 weeks, which is in line with ASCO recommendations [27]. In the study of Mahmood *et al.*, most patients were treated with glucocorticoids with a mean time from admission to steroid initiation of 21.4 ± 16 h [7]. The median equivalent dose of methylprednisolone was 120 mg (range 0 to 1000 mg) and higher doses of steroids were associated with lower peak and discharge troponin levels and lower adverse cardiac events. Other immunosuppression therapies were also administered in a few cases including intravenous immunoglobulin, antithymocyte globulin, and infliximab [7]. According to the review by Palaskas *et al.*, re-initiation of ICI therapy is generally not recommended [23]. Patients should also be treated with conventional cardiac therapy, bradyarrhythmias, in particular advanced AV block, warrant temporary pacemaker insertion [23].

## Conclusion

In conclusion, we report a rare case of pembrolizumab-induced myocarditis with complete atrioventricular block concomitant with myositis in a metastatic bladder cancer patient. As the spectrum of use of immune checkpoint inhibitors is continuing to rise, oncologists, cardiologists, emergency department physicians, pharmacists, and other specialists should be vigilant for this immune-related adverse event, particularly due to its early onset, challenging assessment and diagnosis, and fulminant progression. Troponin monitoring in high-risk patients, along with baseline TTE may help identify ICIM promptly.

## Data Availability

All data generated or analyzed during this study are included in this published article.

## Abbreviations

ICI: Immune checkpoint inhibitor
CTLA-4: Cytotoxic T lymphocyte-associated antigen 4
PD-1: Programmed cell death protein 1
PD-L1: Programmed cell death ligand 1
irAEs: Immune-related adverse events
ICIM: Immune checkpoint inhibitor-associated myocarditis
AV: Atrioventricular
ECG: Electrocardiogram
TTE: Transthoracic echocardiogram
LVEF: Left ventricular ejection fraction
AChR-Ab: Acetylcholine receptor binding antibodies
MACE: Major cardiac event
